# Hypertension Prevalence in Delhi, India: Socioeconomic Disparities Among Adults Aged 19 to 55

**DOI:** 10.7759/cureus.93418

**Published:** 2025-09-28

**Authors:** Vasundhra Chand, Neena Bhatia, Gurdayal Toteja

**Affiliations:** 1 Food and Nutrition and Food Technology, Lady Irwin College, University of Delhi, New Delhi, IND; 2 Jodhpur City Knowledge and Innovation Foundation, Indian Institute of Technology, Jodhpur, IND

**Keywords:** adults, delhi, hypertension, prevalence, socioeconomic groups

## Abstract

Introduction: Hypertension is a critical modifiable risk factor for non-communicable diseases (NCDs), contributing significantly to premature death and morbidity. There is limited data on hypertension prevalence in Delhi. Hence, the study was conducted to assess the prevalence of hypertension among adults.

Methods: A cross-sectional study was conducted among 930 adults aged 19 to 55 years in Delhi, in a population subgroup belonging to three income groups (urban slum, low-income group (LIG), middle-income group (MIG)) with 310 participants in each group. After obtaining informed consent, data was collected through interviews with pretested semi-structured questionnaires. Blood pressure was measured three times using a digital machine (OMRON, Kyoto, Japan), and the average reading was used. Data analysis was performed using Microsoft Excel (Redmond, WA, USA) and SPSS (IBM Corp., Armonk, NY, USA), to identify significant associations.

Results: The overall median systolic blood pressure (SBP) was 124 mmHg (interquartile range: 115-133 mmHg), while the median diastolic blood pressure (DBP) was 78 mmHg (interquartile range: 72-86 mmHg). The prevalence of hypertension was 12.2% (n = 113), with a higher proportion in males (15.3%, n = 71) compared to females (9.0%, n = 42), and in the age group 41-55 years (22%, n = 80) compared to 19-40 years (5.7%, n = 33). The overall prevalence of prehypertension was 54.3% (n = 505), stage I hypertension was 11.1% (n = 103), and stage II hypertension was 1.1% (n = 10). MIG adults had the highest prevalence of prehypertension (61.6%, n = 191), followed by LIG (55.5%, n = 172) and urban slum (45.8%, n = 142). Age ≥40 years was strongly associated with prehypertension (OR = 3.24, *p* < 0.001) and hypertension (OR = 6.86, *p* < 0.001). Males had higher odds of prehypertension and hypertension (OR = 2.47 and 3.92, *p* = 0.010 and <0.001). Skilled workers had higher prehypertension odds (OR = 3.82, *p* < 0.001), while middle-income individuals had lower hypertension odds (OR = 0.09, *p* = 0.047).

Conclusion: The study highlights the prevalence of hypertension, emphasizing the need for targeted interventions to address modifiable risk factors.

## Introduction

Hypertension, a significant global public health challenge, is associated with increased risks of cardiovascular diseases, stroke, and mortality. The prevalence of hypertension has been rising due to urbanization, lifestyle shifts, and socioeconomic gaps. Elevated blood pressure (BP) increases the risk of stroke and heart disease, including heart failure and kidney problems [1,2]. Elevated blood pressure is a well-established modifiable risk factor for adverse cardiovascular outcomes, including heart failure and cerebrovascular events. Globally, in 2019, hypertension affected approximately 1.28 billion adults aged 30 to 79 years, with a prevalence rate of 32% among women and 34% among men [3]. Notably, the prevalence of hypertension is increasing in low- and middle-income countries (LMICs) while stabilizing or declining in high-income countries [4]. Between 1990 and 2019, the global prevalence of hypertension among individuals aged 30-79 years nearly doubled, increasing from 331 million to 626 million in women and from 317 million to 652 million in men [5]. In India, 28.1% of adults were reported to have hypertension [6], with a lack of awareness being a significant contributing factor. Often, hypertension remains undetected until complications such as cardiovascular disease or stroke occur. Globally, hypertension is a leading cause of mortality, responsible for 38.1% of ischemic stroke deaths and 42.5% of hemorrhagic stroke deaths [7]. 

Several epidemiological studies indicated a rapid increase in hypertension burden across various states in India [8,9]. Hypertension prevalence in India increased from 18.1% in National Family Health Survey (NFHS)-4 (2015-2016) to 22.8% in NFHS-5 (2019-2021) [10]. Despite heightened awareness efforts, hypertension control remains low, with less than 20% of affected individuals achieving effective management [5]. Therefore, this community-based study aims to estimate the prevalence of hypertension among adults aged 19 to 55 years in Delhi, India, and to examine associated socioeconomic disparities. The findings are intended to inform targeted public health interventions for early detection and effective management of hypertension.

## Materials and methods

Study design and setting

This study is part of a broader, unpublished research project titled “Assessment of Dietary Pattern, Nutritional Status, Metabolic and Behavioural Risk Factors of Non-Communicable Diseases among the Adult Population of West Delhi,” conducted at Lady Irwin College, University of Delhi, New Delhi, India. However, in this article, we present a focused analysis titled “Hypertension Prevalence in Delhi, India: Socioeconomic Disparities Among Adults Aged 19 to 55."

The community-based cross-sectional study was conducted between August 2021 and September 2022 in selected urban localities of Delhi, India. The study targeted adults aged 19-55 years, a working-age population increasingly vulnerable to early-onset hypertension due to urban lifestyle factors such as stress, poor diet, and sedentary behaviour. This age group is particularly important for early detection and prevention strategies and was selected for its relevance and feasibility within community-based settings.

Study population and site selection

Participants were recruited from three clearly defined income groups: urban slum (US) - Kamla Nehru Camp, Kirti Nagar (west Delhi); low-income group (LIG) - J.J. Colony, Pappankalan (southwest Delhi); and middle-income group (MIG) - Mahavir Enclave, Dwarka Sector 1 (southwest Delhi). Household classification was based on class and type of houses as defined by the Indian Council of Medical Research (ICMR) [11].

Eligibility criteria

Inclusion criteria were adults aged 19-55 years, residents of the selected localities residing for more than one year, and willing to provide informed consent. Exclusion criteria were individuals unwilling to participate, seriously ill patients, pregnant or lactating women, and individuals on any medications including antihypertensives or weight-loss drugs.

Participant selection

Participants were recruited using purposive area sampling to select study sites based on predefined socioeconomic classifications (US, LIG, and MIG), followed by snowball sampling within each site to identify eligible adults. This approach enabled effective coverage of diverse urban populations, including those in densely populated or hard-to-reach areas.

Sample size calculation

The sample size was calculated using the formula N = z2p(1-p) x Design effect/d2, where z = 1.96 (95% confidence level); p = 0.16 (prevalence of NCDs from a previous study) [12]; d = 0.05 (absolute precision); and Design effect = 1.5. The total sample size was 930 adults, with 310 participants from each socioeconomic group.

Data collection tools and procedures

Data were collected through a semi-structured questionnaire designed by the authors, drawing on a thorough literature review and field experience. The tool incorporated elements from the WHO STEPS questionnaire and adapted items from prior Indian studies on hypertension and lifestyle risk factors [13,14]. Socioeconomic status was assessed using the 2022 update of the Modified Kuppuswamy Scale [15]. The tool was pre-tested on a sample of 20 individuals from the target population to assess its clarity, consistency, and relevance. Feedback from the pilot phase was used to refine the wording and structure of the instrument for improved validity and ease of administration. Prior to the commencement of data collection, local community leaders and respected citizens were engaged to seek their cooperation in the study. They were informed about the study objectives, and their support was enlisted to ensure effective communication and smooth coordination with the community. The questionnaire comprehensively assessed socioeconomic and demographic characteristics, lifestyle behaviors (including diet, physical activity, tobacco, and alcohol use), personal and family medical history, anthropometric, biophysical, and biochemical parameters, as well as dietary patterns and nutrient intake. The complete Case Record Form and Questionnaire used for data collection is available in the Appendices.

Blood pressure measurement protocol

Blood pressure was measured using a validated digital device (model HEM-8712; Omron, Kyoto, Japan), employing the oscillometric method. It is known for its ease of use, cost-effectiveness, reliability, and suitability for community-based screenings. A medium cuff size (22-32 cm) was used, suitable for most adult arms. The device features automatic inflation and complies with standard accuracy specifications (±3 mmHg for pressure). Measurements were taken on the left arm (or right arm if the left was injured) after the participant had rested in a seated position with back support, feet flat on the ground, and the arm supported at heart level. Three readings were recorded at five-minute intervals, and the mean value was used for analysis [16]. The device was regularly calibrated to maintain measurement accuracy throughout the study period. Hypertension was classified according to Joint National Committee (JNC) VIII guidelines (Table 1), with systolic BP ≥140 mmHg and/or diastolic BP ≥90 mmHg [17].

**Table 1:** Classification of blood pressure (BP) Classification of blood pressure based on systolic and diastolic values measured in millimetres of mercury (mmHg), as per the Joint National Committee (JNC) VIII guidelines [17]. This table is adapted from publicly available guidelines; no permission was required to reproduce this data.

Blood Pressure classification	Systolic BP (mmHg)	Diastolic BP (mmHg)
Normal	< 120	< 80
Pre-hypertension	120 – 139	80 – 89
Stage I Hypertension	140 – 159	90 – 99
Stage II Hypertension	≥ 160	≥ 100

Ethical considerations

Ethical clearance was obtained from the Institutional Ethics Committee of Lady Irwin College, University of Delhi. Participants were informed about the study objectives using a participant information sheet, and written informed consent was obtained before data collection. Adequate time was provided for participants to consider their involvement, and it was emphasized that participation was entirely voluntary, with the right to withdraw at any time without any consequences.

All collected data were kept strictly confidential, anonymized during handling, and securely stored with access limited to authorized study personnel. The study adhered to the Declaration of Helsinki (1964, with amendments) and International Conference on Harmonisation (ICH) good clinical practice (GCP) guidelines, ensuring ethical integrity with respect to beneficence, respect for persons, and justice.

Data management

All data collected from questionnaires and blood pressure measurements were reviewed daily to ensure completeness and accuracy. Interview schedules were checked immediately after each interview for consistency. Data were then cleaned, coded, and entered into a Microsoft Excel (Redmond, WA, USA) spreadsheet. Double data entry and validation procedures were implemented to maintain accuracy and minimize errors. All identifying information was anonymized to ensure participant confidentiality throughout the data handling process.

Statistical analysis

Data were analyzed using SPSS Version 25.0 (IBM Corp., Armonk, NY, USA). Descriptive statistics (mean, median, and standard deviation) were used to describe continuous variables, while frequencies and percentages were used to describe categorical variables. Chi-square test, T-test, Kruskal-Wallis Test, Freeman-Halton Test, ANOVA and multinomial logistic regression were used, with the significance level set at 0.05.

## Results

Data were collected from 930 adults across three clusters: Kirti Nagar (US), Pappankalan (LIG), and Dwarka Sec.-1 (MIG), with equal gender representation in each location (155 males and 155 females).

Characteristics of the study population

At the household level, a higher proportion of Hindu families was found in MIG (99.3%) compared to LIG (92.2%) and US areas (75.1%) households (p < 0.001). Caste distribution varied significantly across residential groups. The majority of families in the US and LIG categories belonged to the combined Scheduled Castes (SC)/Scheduled Tribes (ST)/Other Backward Classes (OBC) groups, with only 29.5% classified as General, while MIG households (89.9%) were predominantly from the General category (p < 0.001). Based on income tertiles, nearly all US households (91.9%) belonged to the lowest income group, most LIG households (89.2%) fell into the middle income group, while all MIG households (100%) were in the highest income group (p < 0.001) (Table 2).

**Table 2:** Household and Individual level demographic characteristics of adults Data are represented as number (n) and percentage (%). Differences with p < 0.05 were considered statistically significant. a = Kruskal-Wallis Test, b = Freeman-Halton Test, Chi-Square Test; LIG = Lower Income Group; MIG = Middle Income Group; SC = Scheduled Castes; ST = Scheduled Tribes; OBC = Other Backward Classes

Category (Household level)		Urban Slum (N = 173)	LIG (N = 166)	MIG (N = 139)	Total (N = 478)	p-value
		n (%)	n (%)	n (%)	n (%)	
Religion						
Hindu		130 (75.1)	153 (92.2)	138 (99.3)	421 (88.1)	< 0.001
Muslim		43 (24.9)	13 (7.8)	1 (0.7)	57 (11.9)	
Caste						
SC		53 (30.6)	58 (34.9)	4 (2.9)	115 (24.1)	< 0.001
ST		11 (6.4)	30 (18.1)	3 (2.2)	44 (9.2)	
OBC		58 (33.5)	29 (17.5)	7 (5.1)	94 (19.7)	
General		51 (29.5)	49 (29.5)	125 (89.9)	225 (47.1)	
Monthly Family Income (in Rupees) [Median (Q1 - Q3)]^a^		10000 (8000 - 12000)	28000 (22000 - 35000)	120000 (90000 - 150000)	25000 (12000 - 83750)	< 0.001
Family Income (Based on tertiles)						
Low (500-15000)		159 (91.9)	6 (3.6)	0 (0)	165 (34.5)	< 0.001
Middle (15000-39640)		14 (8.1)	148 (89.2)	0 (0)	162 (33.9)	
High (39640-270000)		0 (0)	12 (7.2)	139 (100)	151 (31.6)	
Category (Individual level)	Urban Slum (N = 310)		LIG (N = 310)	MIG (N = 310)	Total (N = 930)	p-value
	n (%)		n (%)	n (%)	n (%)	
Age						
19-40 years		209(67.4)	188(60.6)	170(54.8)	567 (60.9)	< 0.01
41-55 years		101(32.6)	122(39.4)	140(45.2)	363 (39.1)	
Education^b^						
Illiterate	79 (25.5)		26 (8.4)	10 (3.2)	115 (12.4)	< 0.001
Only read	3 (1)		0 (0)	0 (0)	3 (0.3)	
Can read and write	58 (18.7)		46 (14.8)	0 (0)	104 (11.2)	
Primary school or literate	42 (13.6)		44 (14.2)	6 (1.9)	92 (9.9)	
Middle school certificate	40 (12.9)		59 (19)	14 (4.5)	113 (12.2)	
High school certificate	43 (13.9)		57 (18.4)	20 (6.5)	120 (12.9)	
Intermediate or post high school diploma	23 (7.4)		51 (16.5)	59 (19)	133 (14.3)	
Graduate	21 (6.8)		27 (8.7)	124 (40)	172 (18.5)	
Postgraduate	1 (0.3)		0 (0)	77 (24.8)	78 (8.4)	
Occupation						
Unemployed	99 (31.9)		121 (39)	137 (44.2)	357 (38.4)	< 0.001
Unskilled fixed income	115 (37.1)		86 (27.7)	3 (1)	204 (21.9)	
Unskilled w/o fixed income	14 (4.5)		12 (3.9)	3 (1)	29 (3.1)	
Skilled fixed income	68 (21.9)		67 (21.6)	140 (45.2)	275 (29.6)	
Skilled w/o fixed income	14 (4.5)		24 (7.7)	27 (8.7)	65 (7)	

At the individual level, the proportion of adults aged 19-40 years was higher in US (67.4%), while older adults aged 41-55 years were more in MIG (45.2%) (p < 0.01). Educational attainment was lowest in the US group, which had the highest proportion of illiterate adults (25.5%). In contrast, MIG adults exhibited the highest education levels, with only 3.2% illiterate, 40% holding graduate degrees, and 24.8% postgraduates (p < 0.001). In terms of occupation, unskilled employment was more common in US (37.1%), whereas skilled occupations were reported more frequently among MIG adults (45.2%) (p < 0.001) (Table 2).

Distribution of systolic and diastolic blood pressure

The overall median systolic blood pressure (SBP) was 124 mmHg (IQR: 115-133), with a mean of 124.9 ± 16.5 mmHg, while the median diastolic blood pressure (DBP) was 78 mmHg (IQR: 72-86), with a mean of 88.2 ± 5.9 mmHg (Table 3).

**Table 3:** Blood pressure measurements according to socio-economic groups, gender and age groups. Test statistics and p-values are reported for comparisons within socio-economic status (F-test), gender (T-test), and age groups (T-test). *F-test; #T-test; Differences with p < 0.05 were considered statistically significant and p < 0.0001 highly significant. SBP = Systolic Blood Pressure; DBP = Diastolic Blood Pressure; SD = Standard Deviation; LIG = Lower Income Group; MIG = Middle Income Group.

Type of population	SBP (mmHg)			DBP (mmHg)		
	Median (Q1 - Q3)	Mean ± SD	Test statistics (p-value)	Median (Q1 - Q3)	Mean ± SD	Test statistics (p-value)
Socio-economic groups						
Urban slum (n=310)	120 (112-129)	121.7 ± 15.8	6.25* (<0.001)	77 (69.3-84)	77.4 ± 11.2	4.88* (0.008)
LIG (n=310)	124 (115-132)	124.1 ± 16.8		79 (72-87)	79.9 ± 10.5	
MIG (n=310)	126 (119-135)	128.8 ± 16.1		79 (72-86)	79.4 ± 9.9	
Gender						
Male (n=465)	126(119-135)	128.2 ± 15.8	6.25^#^ (<0.05)	80(74-88)	80.9 ± 10.7	6.01^#^ (0.009)
Female (n=465)	121(111-130)	121.7 ± 15.9		76(70-84)	76.8 ± 10.1	
Age groups						
19-40 years (n=567)	121 (112-128)	121.9 ± 13	8.4^#^ (<0.001)	86 (78-93)	85.5 ± 12.4	0.24^#^ (0.81)
41-55 years (n=363)	128 (120-140)	131.3 ± 18.5		85 (78-93)	85.7 ± 12.2	
Overall (n=930)	124 (115-133)	124.9 ± 16.5		78 (72-86)	88.2 ± 5.9	

Socioeconomic disparities in blood pressure levels

The median (126 mmHg) and mean (128.8 mmHg) levels of SBP were highest in the MIG group, followed by the LIG and US groups. In the US group, the median and mean levels of SBP were 120 mmHg and 121.7 mmHg, respectively. The mean difference in SBP across the socioeconomic groups was statistically significant (p<0.0001). The median level of DBP was the same (79 mmHg) in both the MIG and LIG groups, while it was 77 mmHg for adults in the US. Similarly, the mean level of DBP was the same (79 mmHg) in the MIG and LIG groups, while it was 77.4 mmHg in the US group. The mean difference was statistically significant (p<0.05) (Table 3).

Gender differences in blood pressure

The median level of SBP was higher in males (126 mmHg) compared to females (121 mmHg). The mean level of SBP in males was also higher (128.2 mmHg) compared to females (121.7 mmHg), a statistically significant difference (p<0.05). The median level of DBP in males and females was 80 mmHg and 76 mmHg, respectively. The mean level of DBP was higher in males (80.9 mmHg) than in females (76.8 mmHg) and the difference was found to be statistically significant (Table 3).

Age-related variations in blood pressure

The median and mean levels of SBP and DBP were analyzed in two age groups: 19-40 years (n=567) and 41-55 years (n=363). The median level of SBP was higher (128 mmHg) in the 41-55 years age group compared to adults aged 19-40 years (121 mmHg). The mean level of SBP also showed similar trends, with the 41-55 years age group having a mean SBP of 131.3 mmHg, while the 19-40 years age group had a mean SBP of 121.9 mmHg. This difference was statistically significant (p<0.001). There was not much difference in the median and mean levels of DBP between the two age groups. The older age group had a median DBP of 86 mmHg, while the younger age group had a median DBP of 85 mmHg. Similarly, the mean DBP was 85.7 mmHg and 85.5 mmHg in the 19-40 years and 41-55 years age groups, respectively (Table 3).

Prevalence of hypertension and pre-hypertension

The prevalence of stage I hypertension (140-159/90-99 mmHg) was highest in the MIG group (15.2%), followed by the LIG (9.4%) and US (8.7%) groups. The prevalence of stage II hypertension (≥160/100 mmHg) was less than 2% in all socioeconomic groups. Similarly, the prevalence of pre-hypertension was higher in the MIG group (61.6%), followed by the LIG (55.5%) and US (45.8%) groups. The difference was statistically significant (p<0.05). The prevalence of hypertension (≥140/≥90 mmHg) was higher in males (15.3%) than in females (9%). Similarly, pre-hypertension was more prevalent among males (60.4%) compared to females (48.3%). Adults aged 41-55 years had a higher prevalence of hypertension (19.5%) than the younger group (5.6%). This difference was statistically significant (p<0.05) (Table 4).

**Table 4:** Blood pressure levels among adults according to socio-economic groups, gender and age groups Data are represented as number (N) and percentage (%). Differences with p < 0.05 were considered statistically significant and p < 0.0001 highly significant. Chi-square test used to compare blood pressure categories across socio-economic groups, gender, and age groups. LIG = Lower Income Group; MIG = Middle Income Group.

Type of population	Blood pressure (mmHg)					
	Normal (< 120/80)	Pre-hypertensive (120-139/80-89)	Stage I hypertension (140-159/90-99)	Stage II hypertension (>= 160/100)	χ 2 statistic	p-value
	n(%)	n(%)	n(%)	n(%)		
Socio-economic groups						
Urban slum (n=310)	138(44.5)	142(45.8)	27(8.7)	3(1.0)	38.4	<0.00001
LIG (n=310)	104(33.5)	172(55.5)	29(9.4)	5(1.6)		
MIG (n=310)	70(22.6)	191(61.6)	47(15.2)	2(0.6)		
Gender						
Male (n=465)	113(24.3)	281(60.4)	63(13.6)	8(1.7)	38.8	<0.00001
Female (n=465)	199(42.7)	224(48.3)	40(8.6)	2(0.4)		
Age groups						
19-40 years (n=567)	262 (46.2)	274 (48.3)	32 (5.6)	1 (0.1)	129.1	<0.05
41-55 years (n=363)	50 (13.8)	233 (64.2)	71 (19.5)	9 (2.5)		
Overall (n=930)	312(33.5)	505(54.3)	103(11.1)	10(1.1)		

The multivariate analysis identifies key factors significantly associated with prehypertension (PHTN) and hypertension (HTN). Adults aged ≥40 years had significantly higher odds of both PHTN (OR = 3.24, 95% CI = 1.76-5.95, p < 0.001) and HTN (OR = 6.86, 95% CI = 3.70-12.7, p < 0.001) compared to younger adults. Males were also at greater risk, with higher odds of PHTN (OR = 2.47, 95% CI = 1.24-4.93, p = 0.010) and HTN (OR = 3.92, 95% CI = 1.94-7.91, p < 0.001) than females. Muslim adults had significantly higher odds of PHTN (OR = 2.92, 95% CI = 1.16-7.34, p = 0.022) and HTN (OR = 2.53, 95% CI = 0.98-6.56, p = 0.045) compared to Hindus. Education appeared protective, with graduates having significantly lower odds of HTN (OR = 0.26, 95% CI = 0.07-0.90, p = 0.034). Skilled workers had higher odds of PHTN (OR = 3.82, 95% CI = 1.76-8.28, p < 0.001), indicating potential occupational stress. Middle-income individuals showed reduced odds of HTN (OR = 0.09, 95% CI = 0.01-0.97, p = 0.047) compared to the low-income group. No significant associations were found for caste, residential area, tobacco use, or alcohol consumption. Overall, age, gender, religion, education, occupation, and income emerged as important predictors of elevated blood pressure (Table 5).

**Table 5:** Multinomial Logistic Regression Analysis of Factors Associated with Prehypertension and Hypertension Compared to Normotension* LIG = Lower Income Group; MIG = Middle Income Group ; OR = odds ratio; CI = Confidence Interval at 95%; HTN = Hypertension; PHTN = Prehypertension. Differences with p < 0.05 were considered statistically significant and p < 0.0001 highly significant. *The reference category is normotension (normal blood pressure)

Characteristics	Category	PHTN			HTN		
		OR	95% CI	p-value	OR	95% CI	p-value
Age [ref: <40 years]	>=40 years	3.24	1.76-5.95	< 0.001	6.86	3.70- 12.7	< 0.001
Religion [ref: Hindu]	Muslim	2.92	1.16-7.34	0.022	2.53	0.98-6.56	0.045
Caste [ref: General]	SC	1.85	0.77-4.44	0.2	1.15	0.51-2.57	0.7
	ST	1.67	0.55-5.07	0.4	0.41	0.10-1.61	0.2
	OBC	0.98	0.41-2.36	0.9	0.43	0.18-1.02	0.057
Gender [ref: Female]	Male	2.47	1.24-4.93	0.010	3.92	1.94-7.91	< 0.001
Education [ref: Illiterate]	Upto 12^th^	0.81	0.28-2.37	0.7	0.42	0.17-1.04	0.060
	Graduate or more	0.77	0.20-2.98	0.7	0.26	0.07-0.090	0.034
Occupation [ref: Unemployed]	Unskilled	1.23	0.53-2.86	0.6	0.85	0.39-1.86	0.7
	Skilled	3.82	1.76-8.28	< 0.001	0.98	0.46-2.10	0.9
Income [ref: Low]	Middle	4.61	0.24-89	0.3	0.09	0.01-0.97	0.047
	High	2.16	0.21-22.3	0.5	0.42	0.07-2.43	0.3
Cluster [ref: Urban Slum]	LIG	4.83	0.74-31.5	0.10	0.66	0.15-2.90	0.6
	MIG	11.7	0.54-25.5	0.5	0.56	0.05-5.85	0.6
Tobacco [ref: Never consumed]	Current user	0.66	0.30-1.45	0.3	1.21	0.59-2.51	0.6
Alcohol [ref: Never consumed]	Current user	0.71	0.28-1.80	0.5	1.43	0.61-3.32	0.4

**Figure 1:**
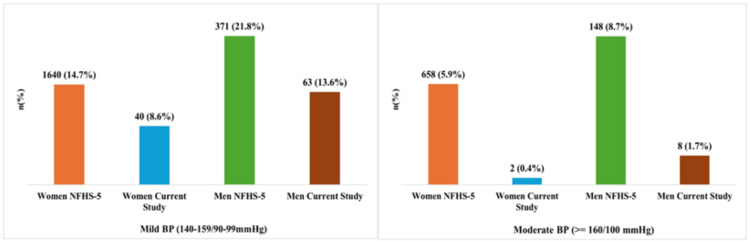
Comparison of hypertension prevalence among women and men in Delhi based on National Family Health Survey-5 (NFHS-5) and the current study. Total participants: Delhi NFHS-5 survey [18] (N = 12,859; Women: 11,159, Men: 1,700); Current Study (N = 930; Women: 465, Men: 465). Values are presented as number of participants with percentages in parentheses: N (%). BP = blood pressure

The data illustrate the differences in the prevalence of mild and moderate hypertension among women and men in Delhi as per the NFHS-5 (2019-2021) with the current study. The prevalence of mild hypertension (140-159/90-99 mmHg) as per the NHFS survey in Delhi was 14.7% among women and 21.8% among men, while moderate hypertension (≥160/100 mmHg) affected 5.9% of women and 8.7% of men. In contrast, the present study reported significantly lower figures, with 8.6% of women and 13.6% of men showing stage I hypertension, and only 0.4% of women and 1.7% of men exhibiting stage II hypertension (Figure 1).

## Discussion

The overall prevalence of hypertension and pre-hypertension in the study was 12.2% and 54.3%, respectively. Notably, the prevalence of hypertension was highest among the MIG (15.8%) followed by LIG (11%) and US (9.7%) groups.

A study conducted in urban Varanasi (n=640, aged 25-64 years) reported a similar trend, with higher hypertension prevalence in the upper-income group (30%) compared to the lower (21.4%) and lower-middle groups (20.2%) [19]. Another cross-sectional study of adults aged 20-40 years in an urban area of Karnataka found a hypertension prevalence of 17.9%, with the highest rates in Class II SES (23.7%), followed by Class IV (21.4%) and Class V (14.6%) [20]. In a community-based study involving 423 adults aged 18-59 years in a West Delhi urban slum, the overall hypertension prevalence was 25.3%, with higher rates in males (27.9%) than females (22.8%) and the highest prevalence in the 46-59 years age group (43.0%). The prevalence of stage I and stage II hypertension was 16.1% and 9.2%, respectively [21]. A 2019 study conducted in urban areas of Lucknow among 300 adults aged over 18 years reported an overall hypertension prevalence of 14.6% [22]. 

The findings of the current study align with previous research, including a repeated cross-sectional analysis using NFHS data [23]. Compared to national data from the NFHS surveys, our study shows a somewhat lower hypertension prevalence than NFHS-5 (18.3%) [24]. The variation may be due to differences in sampling frames, age ranges, urban focus, and measurement protocols. Varghese et al. reported a similar prevalence of hypertension among adults aged 18-98 years across various regions of the country, with a higher proportion (30.6%) in males than in females (25.7%) [6]. Broadening the comparison to a global context reveals much higher prevalence rates in Western countries. For instance, according to the U.S. National Health and Nutrition Examination Survey (NHANES) data collected from August 2021 to August 2023, the hypertension prevalence among adults aged ≥18 years was estimated at 47.7%, with 50.8% in men and 44.6% in women [25]. Similarly, the Health Survey for England (2019) found that approximately 30% of adults had high blood pressure - with about 14% of men and 11% of women having untreated hypertension - indicating overall prevalence rates that exceed 25-30% [26]. In Shenzhen, China, a study of adults aged 18-69 years found that hypertension rates increased from 17.71% in 2009 to 24.01% in 2018 among males (p<0.001), while the prevalence among females was 13.5% [27]. These estimates are substantially higher than those observed in India, likely reflecting differences in lifestyle and health system factors.

Age was identified as a significant factor, with older individuals having higher chances of developing hypertension. The current study observed a higher prevalence of hypertension among those aged 41-55 years (22%) compared to those aged 19-40 years (5.7%). A study carried out among 420 adults in Karnataka reported that the prevalence of hypertension was highest among the 35-40 years age group (27%) and lowest among those aged between 20-24 years (5.3%) [20]. A national survey from January 20, 2015, to December 4, 2016, found a 45.9% hypertension prevalence among those over 45 years [10]. Age-wise prevalence of hypertension showed that the highest (67.2%) prevalence of hypertension was in the age group between 41 and 60 years, and the least prevalence (42.8%) was observed in the age group of ≤20 years [28]. This is in line with other Asian studies where the risk of hypertension increased with age >50 years [29].

This study supports existing Indian research showing a high burden of undiagnosed hypertension. Large-scale studies have reported that among older adults, 42.3% had undiagnosed hypertension, 6% were untreated, and 18.7% were undertreated [30]. Nationally, self-reported and undiagnosed hypertension rates were 27.4% and 17.8%, respectively [31]. The Longitudinal Aging Study in India (LASI) highlights that nearly one in five adults aged 45 and above remain undiagnosed, putting them at increased risk for cardiovascular complications. Although hypertension risk rises significantly with age (OR: 4.6; 95% CI: 3.0-7.1 for ages 60-79 vs. <40), the rates of undiagnosed and untreated cases decline with age [32]. Additionally, in Kerala, younger adults, especially men, had lower awareness, treatment, and control compared to women [33].

In alignment with previous research [22,34], the present study found that adults aged over 40 years, males, and those engaged in skilled occupations had significantly higher odds of both prehypertension and hypertension. Moreover, higher educational attainment, particularly graduation and above, was associated with lower odds of hypertension. These findings reinforce the influence of age, gender, occupation, and education as important determinants of elevated blood pressure.

Despite our efforts, this study has some limitations. Its cross-sectional design limits causal interpretation, and the relatively small sample size may affect generalizability of findings to the broader Indian population. The exclusion of adults over 55 years may have led to an underestimation of overall hypertension prevalence, as the condition is more common in older populations. However, the focus on the 19-55 years age group aligns with national surveillance data and ensures relevance for early intervention strategies. Additionally, the study was limited to urban areas and did not include higher-income groups, which may further limit the applicability of the findings to the broader population.

Strengths of the study

This study employed a community-based approach across varied urban socioeconomic groups, providing valuable insights into hypertension disparities. Use of a validated Omron digital monitor with standardized protocols ensured reliable blood pressure measurements. Focusing on adults aged 19-55 years aligns with priorities for early intervention.

## Conclusions

This study underscores the significant prevalence of hypertension and pre-hypertension in urban Delhi, with 12.2% of adults being hypertensive and 54.3% being pre-hypertensive. The MIG group exhibited the highest rates of pre-hypertension (61.6%) and stage I hypertension (15.2%), while US residents had the highest normal BP (44.5%), and LIG adults had the highest stage II hypertension (1.6%). The high prevalence of hypertension in this area highlights the need for preventive measures to mitigate the risk of chronic diseases. Men were found to be particularly vulnerable, and advancing age was a significant risk factor. The high rate of undiagnosed hypertension in India highlights major gaps in healthcare screening and poses a significant clinical, public health, and economic challenge.

Improved surveillance systems and community-based screening programs are crucial for early detection. Addressing the lack of awareness regarding hypertension status through enhanced health literacy is also imperative. Interventions such as weight management, promotion of physical activity, and dietary improvements are strongly recommended. These targeted interventions can help reduce the burden of hypertension and improve overall health outcomes. Further research should explore the specific lifestyle and environmental factors contributing to these socioeconomic disparities in hypertension prevalence, enabling more tailored and effective public health strategies.

## Appendices

Case record form and questionnaire

https://drive.google.com/file/d/1ItxwnZ8AIKPMKt8mbWnGpR5wMiOFSRlY/view?usp=sharing
